# Refractive changes after cataract removal in infancy: comparing eyes with and without persistent fetal vasculature

**DOI:** 10.1007/s00417-025-06841-6

**Published:** 2025-04-29

**Authors:** Ana Navarrete, Brice Nguedia Vofo, Hadas Mechoulam, Milka Matanis-Suidan, Nur Azem, Ilana Karshai, Ran David, Irene Anteby

**Affiliations:** Department of Ophthalmology, Hadassah-Hebrew University Medical Center, Kalman Ya’Akov Man Street, POB 12000, Jerusalem, 91120 Israel

**Keywords:** Pediatric cataract, Emmetropization, Persistent fetal vasculature, Refractive errors

## Abstract

**Purpose:**

To evaluate the refractive changes after congenital cataract surgery in persistent fetal vasculature (PFV) vs. non-PFV eyes.

**Methods:**

Retrospective study of 75 eyes with PFV or non-PFV congenital cataract, who underwent surgery before age 7 months (unilateral/first operated eye), during 2007–2018 at a tertiary referral center, with follow-up ≥ one-year.

**Results:**

27 eyes (36%) had PFV, 48 were non-PFV cataracts. Mean age (± SD) at surgery in children with PFV was 2.24 ± 1.23 months and 2.44 ± 1.51 months in the non-PFV group. Mean post-operative follow-up was 64.94 ± 34.67 months. 60% of the eyes remained aphakic for the entire follow-up.

In aphakic children, the mean post-operative spherical equivalent (SE) in the PFV eye was + 18.74D, + 15.73D, + 13.88D, + 12.51D, + 11.29D at one-month, one-year, two-years, three-years and five-years respectively. In the non-PFV eye the SE was + 23.00D, + 20.44D, + 17.84D, + 17.52D, + 18.48D at one-month, one-year, two-years, three-years and five-years respectively. During the entire post-operative course, the SE remained less hyperopic in the PFV eyes (*p* < 0.01).

The rate of emmetropization was similar for PFV and non-PFV eyes. Five-years after surgery the mean myopic shift was -6.82 ± 4.32 D in the PFV eyes and -5.47 ± 2.44D in the non-PFV eyes (*p* > 0.05).

The changes in refraction error did not correlate with either presence of glaucoma, secondary cataract, amblyopia or strabismus.

**Conclusion:**

Eyes with PFV have a similar rate of emmetropization as other pediatric congenital cataracts. Interestingly, aphakic PFV eyes have a less hyperopic refraction during one-month and up to five-years after surgery as compared to non-PFV eyes.

**Supplementary information:**

The online version contains supplementary material available at 10.1007/s00417-025-06841-6.

## Key messages

*****What is known***:**

- Persistent fetal vasculature is often associated with cataract and microphthalmia. Little is known about the refractive changes following cataract surgery in PFV eyes.

*****What is new***:**

- Aphakic PFV eyes showed a lower hyperopic refraction compared to non-PFV eyes during the first month up to five years after surgery, contrary to expectations considering a smaller eye.

- Corneal curvature measurements may partly explain the lower hyperopic refraction in PFV eyes, as PFV eyes tend to have steeper corneas.

**Supplementary information:**

The online version contains supplementary material available at 10.1007/s00417-025-06841-6.

## Introduction

The process of emmetropization in children is affected by various factors including axial length (AL) elongation, corneal curvature reduction, and lens growth during childhood. Emmetropization progression [1] can further be impacted by age [2], lens condition [3–5], and glaucoma [6]. The reduction in hyperopia or myopic shift observed in children following cataract removal is primary attributed to axial length elongation, with the most significant changes happening within the first 18 months of life [7, 8].

Refractive changes after unilateral and bilateral congenital cataract surgery exhibit variability [8] in post-operative refraction, and the myopic shift seen after pediatric cataract surgery is typically logarithmic in aphakic eyes [9].

Persistent fetal vasculature (PFV) is a congenital disorder marked by incomplete regression of fetal vasculature [10] in which the normal development of the eye is arrested. This anomaly often results in a retrolental fibrovascular mass that in most cases is accompanied by cataract with varying degrees of microphthalmia [11]. In less severe forms of PFV, functional visual acuity can be obtained after surgical intervention in at least a third of the affected children [12, 13]. However, studies offer limited data on refractive errors following cataract surgery in eyes with PFV [14, 15]. Additionally, there is a lack of comparison between refractive changes after lensectomy in eyes with and without PFV.

This study aimed to describe the emmetropization process after early cataract surgery while comparing PFV with regular congenital cataracts.

## Methods

Retrospective study of all eyes with PFV or non PFV cataract (defined as eyes with an isolated lens opacity), who underwent lensectomy and anterior vitrectomy in children younger than 7 months (unilateral or the first operated eye in bilateral cases), during the years 2007–2018 at a tertiary hospital, and had a follow-up of at least one year. PFV was defined as eyes with incomplete regression of fetal vasculature including a spectrum of presentations ranging from anterior to posterior findings such as retrolental opacity, elongated ciliary processes, cataract, microphthalmos, a stalk of tissue extending from the optic nerve to the retrolental area, and retinal dysplasia. Exclusion criteria were surgery after age 7 months, follow-up of less than 1 year after lensectomy, incomplete refraction data, and associated anterior segment dysgenesis or ocular pathologies other than PFV. Lensectomy was performed through a limbal incisions with posterior capsulotomy and anterior vitrectomy, either with or without IOL insertion.

The study adhered to the tenets of the Declaration of Helsinki and was approved by our institutional ethics committee.

Data retrieved from the medical records included: sex, laterality, age at surgery, optical status after surgery (aphakia, primary IOL or secondary IOL), and best-corrected visual acuity (BCVA) in verbal children. Cycloplegic refraction and spherical equivalent (SE) after surgery was also obtained at 1 month, 6 months, 1 year, 2 years, 3 years, 5 years post-operative, and at last follow up. Cycloplegic refraction was performed post-operatively by retinoscopy in all children. In children with aphakia or pseudophakia, retinoscopy was performed without contact lens or glasses (no over-refraction). Microphthalmia was a subjective clinical diagnosis by the operating surgeon as an abnormally small eye [12]. AL was measured intraoperatively by A-scan ultrasound biometry in some of the eyes at time of lensectomy.

The change in post-operative refraction (emmetropization rate or myopic shift) was calculated as the difference between the spherical equivalent (SE) at one month post lensectomy and at one, two, three and five years after surgery.

Data regarding complications was retrieved and included (1) ocular hypertension (OHT), defined as intraocular pressure (IOP) of > 20 mmHg at 2 different visits, not related to topical use of steroids, (2) glaucoma, defined as OHT with increased cup/disc ratio, or retinal nerve fiber layers (RNFL) changes seen on optical coherence tomography (OCT), (3) retinal detachment, (4) strabismus, and (5) secondary cataract. High myopia was defined as SE above − 6.00 diopters (D) without history of OHT or glaucoma, at last follow-up.

Best-corrected visual acuity (BCVA) on a decimal scale was obtained at the 3 years visit in verbal children (LEA or Snellen tests). In non-verbal children, non-quantifiable visual acuity was measured by fixation behavior. BCVA at last follow-up was classified as"poor BCVA"when visual acuity was < 0.1, or no fixation, LP or NLP,"functional BCVA"when visual acuity was 0.1–0.4, partial fixation behavior, and"good BCVA"when visual acuity was ≥ 0.5 or normal fixation behavior [16]. The time of follow-up and age at last follow-up were also obtained.

Categorical variables were recorded using frequencies and percentages. Continuous variables were summarized using mean, median, standard deviation (SD), and range (minimum and maximum).

Statistical analyses were performed using SPSS Statistics, version 25.0 (IBM Corp., Armonk, NY). Data normality was analyzed using the Shapiro–Wilk test. Comparing continuous variables between two independent groups were carried out using the two-sample t-test or the non-parametric Mann–Whitney U test. Testing the change in a continuous variable, between two time points, was performed by applying the paired t-test, whereas testing change over three points or more was performed by applying the repeated measures ANOVA model.

Categorical data was compared using Chi Square test. Parameters of interest were reported with confidence intervals (CI). Statistical significance was defined as *P* < 0.05.

## Results

### Demographic data

Our study included 75 eyes of 75 children. Twenty-seven eyes (36%) had PFV, while the remaining 48 eyes had non PFV cataract. Within the PFV group, 13 eyes had anterior PFV, and 14 eyes had combined PFV. PFV was unilateral in 88.8% of the children. Children with non-PFV cataract were mostly bilateral (89.58%). Both groups were similar in terms of age at diagnosis, surgery and last examination. Seventy-six % of the eyes were followed for three years or longer. Table 1 shows all demographic data, comparing between PFV and non PFV congenital cataract. Axial length (AL) measurements at time of lensectomy was available in six PFV and nine non-PFV eyes. Supplementary Table 1 shows axial length measurements available.

**Table 1:** Demographic Data

	All cases # (%)	PFV # (%)	Non PFV # (%)	*P*-Value
# Eyes	75 (100%)	27 (36%)	48 (64%)	
Sex				
Female	35 (46.7%)	15 (55.5%)	20 (42%)	
Male	40 (53.3%)	12 (44.5%)	28 (58%)	
Laterality				
Unilateral	29 (38.7%)	24 (88.9%)	5 (10.4%)	
Bilateral (first operated included)	46 (61%)	3 (11.1%)	43 (89.6%)	
Microphthalmia	26 (34.7%)	22 (81.5%)	4 (8.3%)	*P* < 0.01 ^a^
Family history of high Myopia	2 (2.66%)	0 (0%)	2 (4.16%)	*P* = 0.05^a^
Age at surgery in months (mean ± SD)	2.37 (± 1.41)	2.24 (± 1.23), (range 1–6)	2.44 ± 1.51, (range 1–7)	*p* = 0.55 +
Age at last follow up in months (mean ± SD)	67.31 (± 34.87) (range 14–152)	67.78, (± 34.66), (range 16–148)	67.05, (± 35.35) (range 14–152)	*P* = 0.90 +
Optical status after surgery				*P* = 0.31^a^
* Immediate post-lensectomy aphakia*	45 (60%)	18 (66.7%)	27 (56.2%)	
* Pseudophakia*	30 (40%)	9 (33.3%)	21 (43.8%)	*P* = 0.37^a^
Primary IOL	12(16%)	2 (7.4%)	10 (20.8%)	
Secondary IOL following post-lensectomy aphakia	18 (24%)	7 (25.9%)	11 (22.9%)	
Mean time from lensectomy to secondary IOL insertion (months)		Mean: 29.85 (SD ± 10.06) Median: 27 Range: 17–45	Mean: 42.09 (SD ± 21.88) Median: 36 months Range: 21–90	*P* = 0.06 +
Follow up data:				
* Time of follow up after surgery (mean months ± SD)*	64.94 (± 34.67), (range 12–150)	65.54 (± 34.85) (Range 14–147)	64.61 (± 34.94) (range 12–150)	P = 0.90 +
* Number of patients with follow up of 3 years*	55 (60%)	19 (70.3%)	36 (75%)	
Aphakia	27 (49%)	11 (58%)	16 (44.4%)	
Pseudophakia	28 (51%)	8 (42%)	20 (55.5%)	
* Number of patients with follow up 5 years and longer*	43 (57.3%)	16 (59.2%)	27 (56.2%)	
Aphakia	20 (46.5%)	10 (62.5%)	10 (37%)	
Pseudophakia	23 (53.5%)	6 (37.5%)	17 (63%)	

+ T test. ^a^Chi Square test

### Surgical outcomes

The eyes were left with aphakia in 66.7% and 56.3% of the cases in the PFV and the non PFV group, respectively [Table 1]. An IOL was implanted as a primary procedure in two PFV and 10 non-PFV eyes, and as a secondary implantation in seven PFV and 11 non-PFV eyes, (*p* = 0.37).

**Fig. 1:**
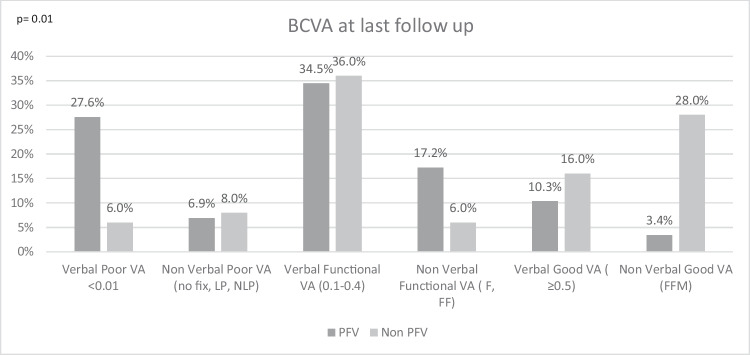
BCVA at last follow up

At 3 years of follow-up 64.6% and 81.48% of the non-PFV and PFV children were able to give a verbal VA. In the non-PFV group, 9.7% remained with poor VA, 67.7% had functional VA and 22.6% achieved good VA. In the PFV group 41.0% had poor VA, 45.4% functional VA and 13.6% reached good visual acuity (*p* = 0.02). Figure 1 shows the BCVA at last follow-up in the PFV and the non-PFV group including both verbal and non-verbal children. Amblyopia was more common in the PFV group.

Other surgical outcomes and complications after surgery are summarized in Table 2.

**Table 2:** Surgical Outcomes and complications

	PFV	Non PFV	P-Value
Secondary OHT/Glaucoma	12 (44.4%)	16 (33.3%)	0.34
*Glaucoma treatment*			
Topical treatment	9	14	
Surgical intervention	3	2	
Resolved at last follow up	8	6	
Remained OHT/Glaucoma	4 (14.81%)	10 (20.83%)	
Visual Acuity last FU			0.01
Poor VA	9 (33.3%)	7 (14.6%)	
Functional VA	14 (51.9%)	19 (39.6%)	
Good VA	4 (14.8%)	22 (45.8%)	
Retinal Detachment	1 (3.7%)	0	0.17
Strabismus	26 (96.3%)	17 (35.4%)	< 0.05
Secondary Cataract	5 (18.5%)	18 (37.5%)	0.08
Time of occurrence after lensectomy (months)	5.5 mean	8.89 mean	0.74

### Post-operative refractive course

#### Subgroup analysis according to optical status

**Fig. 2:**
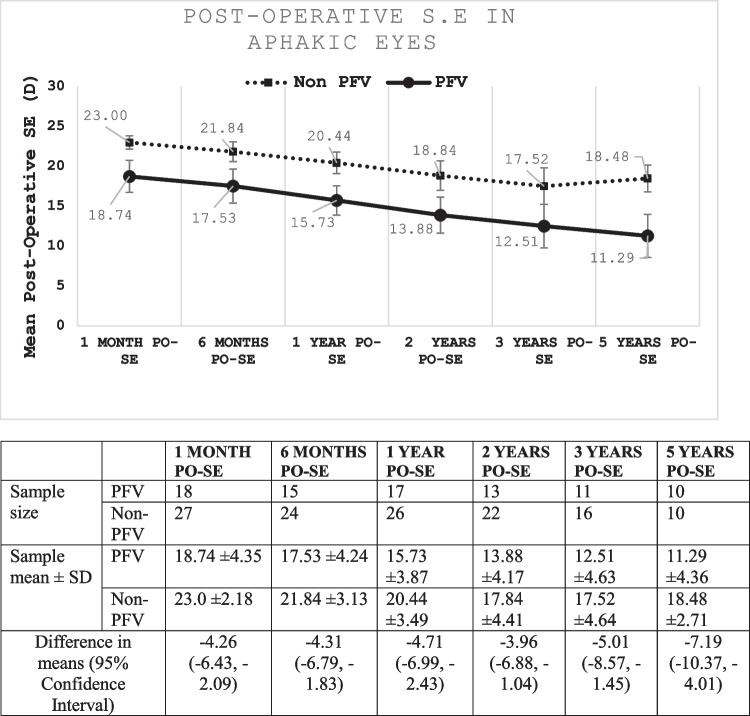
Post-operative S. E in Aphakic eyes

**Fig. 3:**
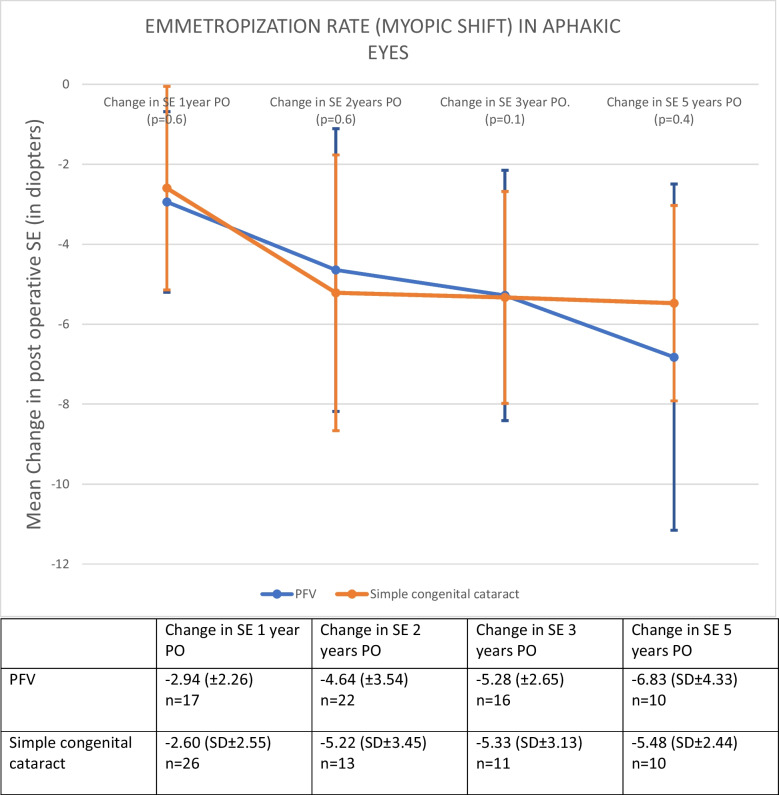
Emmetropization rate (Myopic Shift) in Aphakic eyes

At the time of lensectomy, 60% of the eyes remained aphakic. In aphakic PFV eyes, the post-operative spherical equivalent (SE) exhibited lower hyperopia compared to aphakic non-PFV eyes at various intervals: 1 month, 6 months, 1, 2, 3, and 5 years post-cataract surgery (*p* < 0.01, as illustrated in Fig. 2). Nevertheless, the rate of post-operative refractive change was comparable between the two groups (*P* > 0.05). No significant differences in SE change at 1-, 2-, and 5-years post-operatively were noted between PFV and non-PFV eyes (Fig. 3).

In aphakic eyes, the SE change from the first month to five years post-surgery was − 6.82 ± 4.32 D (95% CI: − 9.51, − 4.14) in PFV eyes and to − 5.47 ± 2.44 D (95% CI:− 6.66, − 3.77) in non-PFV eyes (*p* = 0.56).

**Fig. 4:**
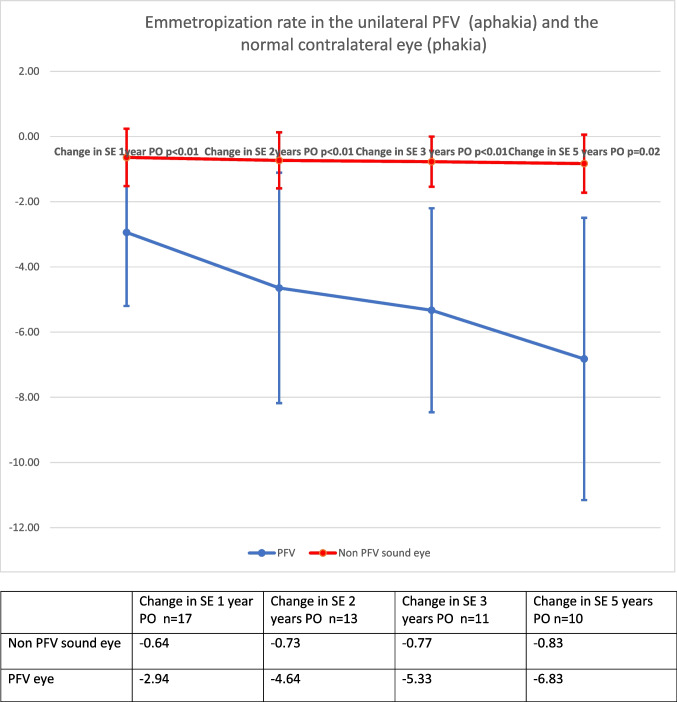
Emmetropization rate in the unilateral PFV (aphakia) and the normal contralateral eye (phakia)

When comparing the change in refraction between the aphakic PFV eye to the normal phakic fellow eye, a more pronounced myopic shift was observed in the PFV eye (Fig. 4) (*p* < 0.01). Five years after cataract removal a myopic shift of − 6.82 ± 4.32 D (95% CI: − 9.51, − 4.14) was evident in the PFV eye, in contrast to a myopic shift of only − 0.83 ± 0.82 D (95% CI 0.24, 1.42) in the fellow eye (*p* = 0.02).

#### Factors that may affect emmetropization after pediatric cataract surgery

Five years after lensectomy, the myopic shift was greater in eyes with primary IOL implantation compared to aphakic eyes, particularly in the non-PFV cases where the refractive change was − 9.96 ± 5.72 D (95% CI − 14.18, − 5.75) in pseudophakic compared to − 5.47 ± 2.44 D (95% CI: − 6.66, − 3.77) in aphakic eyes (*p* < 0.01). In the PFV eyes, the refractive change was − 7.37 ± 1.06 D (95% CI: − 9.46, − 5.29) pseudophakic compared to − 6.82 ± 4.32 D (95% CI: − 9.51, − 4.14) in aphakic eyes (*p* = 0.04); however, while statistically significant, this difference is likely not clinically relevant (Supplementary Figure S1).

Other factors such as high myopia, ocular hypertension, glaucoma, microphthalmia, amblyopia, visual acuity, strabismus, or secondary cataract did not exhibit significant association with refractive changes throughout the follow-up period (See supplementary Table 2).

## Discussion

Persistent fetal vasculature (PFV) is commonly associated with congenital cataract. Remarkably, almost half of all children who undergo surgery for unilateral congenital and infantile cataract in the first 2 years of life and a tenth of the children with bilateral cataract have PFV [13]. Despite this prevalence, the literature concerning refractive outcomes in PFV eyes after cataract surgery remains limited. This study aimed to evaluate long-term refractive changes following cataract surgery in infants with PFV, comparing the outcomes with non PFV eyes.

In our cohort, cataracts associated with PFV and microphthalmia exhibit a similar rate of emmetropization after surgery as eyes with regular (non-PFV) pediatric congenital cataract. The Infantile Aphakia Treatment Study (IATS) study demonstrated marked variability of the myopic shift in aphakic eyes [17]. However, IATS only included eyes with mild forms of PFV, excluding those with severe PFV characteristics such as stretching of ciliary processes, severe microphthalmia or retinal traction [18].

Even though PFV eyes are typically associated with smaller eyes, our study unexpectedly showed a lower hyperopic refraction in aphakic PFV eyes from one month and up to 5 years after surgery when compared to non-PFV eyes. Similarly, Prasad et al. [19], found a low mean post-operative refractive error (SE) in aphakic microphthalmic eyes of 16.85 ± 5.24 and 16.01 ± 1.90 2 weeks and 12 months after surgery, respectively. Prasad et al., did not specifically include eyes with PFV in their study.

The reason for the lower hyperopic refraction found in aphakic eyes with PFV is unclear. This might partly arise from corneal curvature measurements. In the present study AL and keratometry (K) readings were not available in all eyes. It is known that in addition to AL measurements, a lower hyperopic refraction can also be secondary to corneal curvature measurements [20]. Infants’ eyes tend to have steeper keratometry values, with an expected flattening with increasing age [2, 21]. Though limited data exist on keratometry in PFV eyes, many studies suggest that corneas in PFV eyes are steeper, possibly influencing refractive outcomes [2, 21]. Trivedi et al. [2], showed a borderline steeper mean K value in eyes with unilateral cataract in comparison with bilateral cases (*p* = 0.07). Although analysis for PFV was not done, they described a case of PFV eye with keratometry values of 63.5 D [2]. Similarly, Asbell et al. [21] found that in 11 eyes with PFV cataract, the majority had higher keratometry values compared to non-PFV eyes for their specific age group.

Our study looked at different factors that may affect the myopic shift or emmetropization process after pediatric cataract surgery. Notably, primary IOL implantation before 7 months of age led to a greater myopic shift, particularly pronounced in non-PFV eyes where the refractive change was − 9.96 D in pseudophakic vs − 5.47 D in aphakic eyes five years after cataract removal. This aligns with findings in studies like Lambert et al. [4] and Mc Clatchey et al. [22] However, none of these studies included microphthalmic and PFV eyes. Previous studies have shown that when eliminating the refractive effect of the IOL and measuring the refractive change due to the growth of the eye, the rate of refractive growth [9] was similar in aphakic and pseudophakic eyes [4, 22–27]. The greater myopic shift observed in pseudophakic eyes can therefore be the result of an optic effect of the IOL as the eye elongates. The IOL will progressively be farther from the retina as the eye growths, increasing the quantity of myopic shift [22].

Other factors, such as strabismus, microphthalmia, glaucoma, amblyopia and long-term visual outcome, did not significantly impact the emmetropization rate in our study. These observations are consistent with other authors. Lambert et al., [23] did not find a relationship between the presence of OHT/Glaucoma and the rate of refractive growth. However, their study showed a correlation between visual outcomes and refractive changes in eyes that were aphakic [23].

While our study contributes valuable insights into refractive outcomes post-cataract surgery in PFV eyes, it is important to acknowledge its limitations. The retrospective design, absence of axial length and keratometry measurements, and subjective diagnosis of microphthalmia all present potential sources of bias. Wilson et al. [28], emphasize the significance of AL growth in predicting long-term refractive outcomes, particularly the risk of myopic shift, highlighting the importance of monitoring both AL and K values in pediatric cataract cases. Additionally, relying on fixation behavior to assess visual acuity in non-verbal infants, though standard practice in our clinic, may not be as comprehensive as other pediatric ophthalmology methods. Future studies should consider prospective designs that include comprehensive ocular biometrical measurements to address these limitations.

In conclusion, our study stands as the first study that analyzed refractive outcomes after cataract surgery in PFV eyes while comparing them to non-PFV eyes. The study revealed that PFV eyes exhibit a similar rate of emmetropization as other pediatric congenital cataracts cases. Interestingly, aphakic PFV eyes had a less hyperopic refraction during the first month up to five-years after surgery, compared to simple congenital cataract eyes. These insights should be considered when calculating the target IOL power in young infants.

## Supplementary Information

Below is the link to the electronic supplementary material.

ESM 1 (DOCX 45.1 KB)

ESM 2 (DOCX 12.7 KB)

ESM 3 (DOCX 15.0 KB)

## Data Availability

The data that support the findings of this study are available from the corresponding author upon reasonable request.
