# Supplementary material for: Refractive changes after cataract removal in infancy: comparing eyes with and without persistent fetal vasculature
Source: Graefes Arch Clin Exp Ophthalmol. 2025 Apr 29;263(8):2369–77. doi: 10.1007/s00417-025-06841-6 (PMC12414064; doi:10.1007/s00417-025-06841-6)
Supplement: Supplementary file 2 — (DOCX 12.7 KB) [file 417_2025_6841_MOESM2_ESM.docx]

| **Supplementary Table 1. Axial length values available at time of lensectomy** | |
| --- | --- |
| **PFV** | **Axial Length (mm)** |
| Case # 1 | 18.96 |
| Case # 5 | 18.67 |
| Case # 6 | 16 |
| Case # 16 | 18.3 |
| Case # 19 | 17.31 |
| Case #17 | 17 |
|  |  |
| **Non PFV** |  |
| Case # 15 | 18.77 |
| Case #16 | 18.42 |
| Case # 18 | 18.09 |
| Case # 19 | 19.1 |
| Case # 22 | 18.34 |
| Case # 24 | 16.6 |
| Case # 25 | 17.3 |
| Case # 34 | 18.89 |
| Case # 49 | 19.03 |
