# Supplementary material for: Refractive changes after cataract removal in infancy: comparing eyes with and without persistent fetal vasculature
Source: Graefes Arch Clin Exp Ophthalmol. 2025 Apr 29;263(8):2369–77. doi: 10.1007/s00417-025-06841-6 (PMC12414064; doi:10.1007/s00417-025-06841-6)
Supplement: Supplementary file 3 — (DOCX 15.0 KB) [file 417_2025_6841_MOESM3_ESM.docx]

**Supplementary Table 2: Correlation Between Ocular Factors and Changes in Spherical Equivalence Over Follow-Up.**

|  | **Spearman Rho** | **P-value** | **95% Confidence interval** |
| --- | --- | --- | --- |
| **High Myopia** | -0.305 | 0.156 | -0.587, -0.242 |
| **Microphthalmia** | 0.093 | 0.674 | -0.300, 0.467 |
| **Ocular Hypertension** | -0.337 | 0.115 | -0.573, -0.097 |
| **Glaucoma** | -0.138 | 0.531 | -0.538, 0.297 |
| **Strabismus** | -0.302 | 0.161 | -0.635, 0.128 |
| **Amblyopia** | -0.181 | 0.409 | -0.640, -0.337 |
| **Secondary Cataract** | -0.224 | 0.304 | -0.604, 0.210 |
| **Visual Acuity** | 0.101 | 0.647 | -0.326, -0.531 |
