# Supplementary material for: Refractive changes after cataract removal in infancy: comparing eyes with and without persistent fetal vasculature
Source: Graefes Arch Clin Exp Ophthalmol. 2025 Apr 29;263(8):2369–77. doi: 10.1007/s00417-025-06841-6 (PMC12414064; doi:10.1007/s00417-025-06841-6)
Supplement: Supplementary file 3 [file 417_2025_6841_MOESM1_ESM.docx]

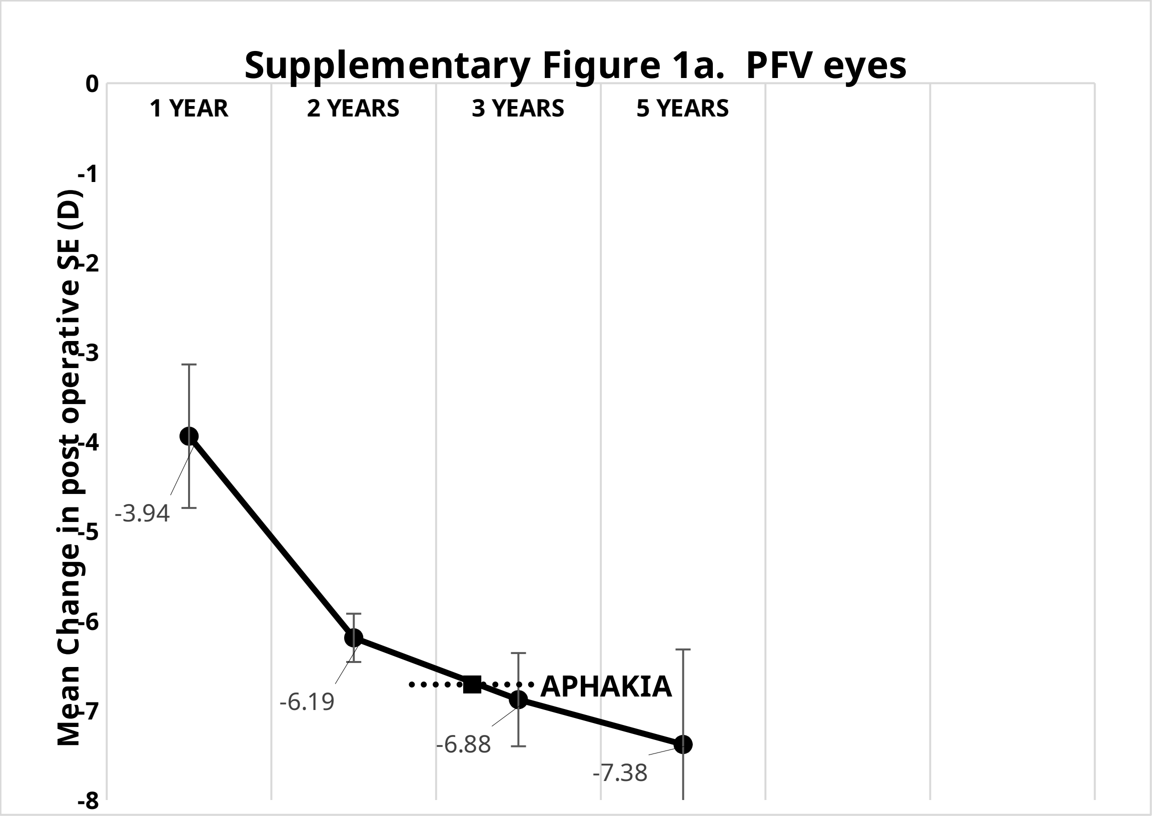
Supplementary Figure 1a. PFV eyes
Mean Change in post operative SE (D)
0
-1
-2
-3
-4
-5
-6
-7
-8
1 YEAR
2 YEARS
3 YEARS
5 YEARS
-3.94
-6.19
-6.88
-7.38
APHAKIA

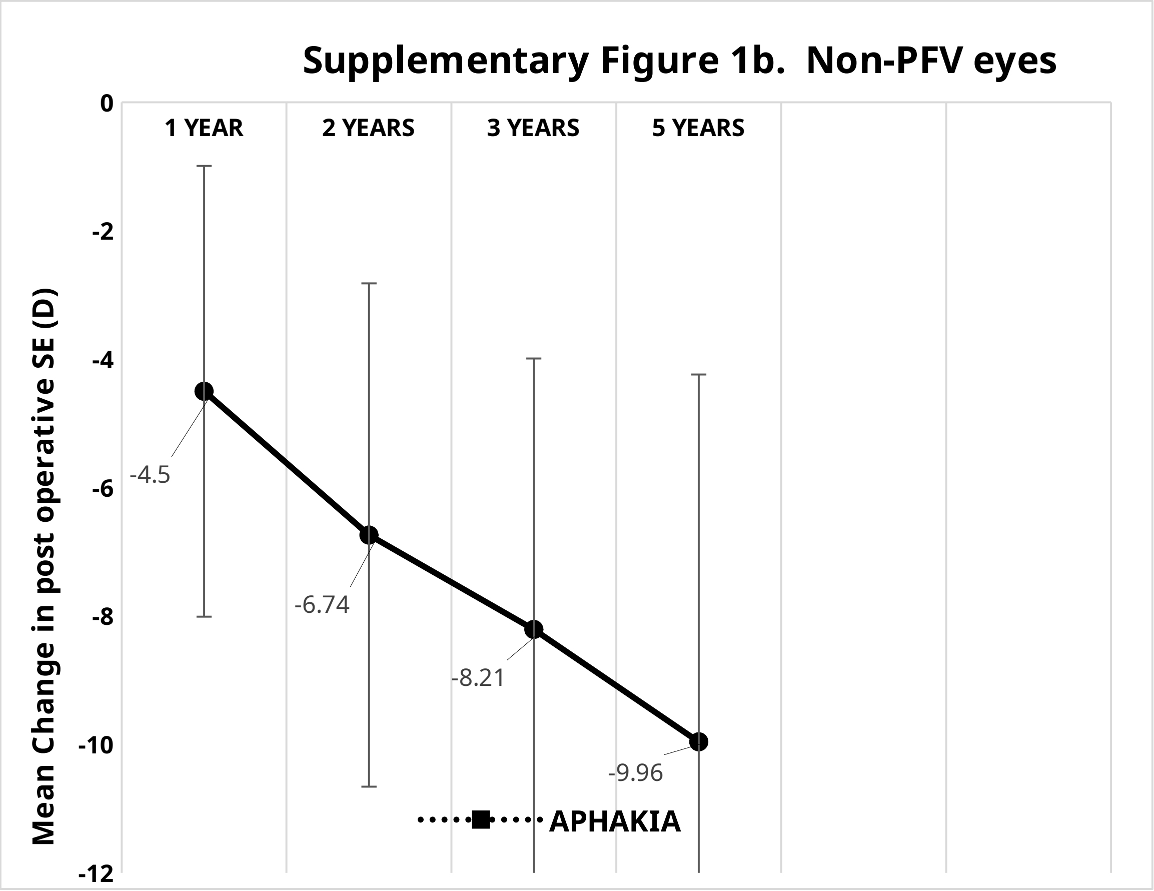
Supplementary Figure 1b. Non-PFV eyes
Mean Change in post operative SE (D)
0
-2
-4
-6
-8
-10
-12
1 YEAR
2 YEARS
3 YEARS
5 YEARS
-4.5
-6.74
-8.21
-9.96
APHAKIA
